# The Association of Metabolic Syndrome with the development of cardiovascular disease among Kazakhs in remote rural areas of Xinjiang, China: a cohort study

**DOI:** 10.1186/s12889-021-10241-w

**Published:** 2021-01-26

**Authors:** Wenwen Yang, Shuxia Guo, Haixia Wang, Yu Li, Xianghui Zhang, Yunhua Hu, Heng Guo, Kui Wang, Yizhong Yan, Jingyu Zhang, Jiaolong Ma, Lei Mao, Lati Mu, Jiaming Liu, Yanpeng Song, Changjing Li, Zhuo Ma, Rulin Ma, Jia He

**Affiliations:** Department of Public Health, Shihezi University School of Medicine, Shihezi, 832000 Xinjiang China

**Keywords:** Metabolic syndrome, Cardiovascular disease, Kazakhs

## Abstract

**Background:**

Metabolic syndrome (MS) can promote the development of cardiovascular disease (CVD). The objective of this study was to examine the association of MS and its components with CVD, to further prevent and control CVD in Kazakhs.

**Methods:**

In the cohort study, a total of 2644 participants completed the baseline survey between April 2010 and December 2012.The follow-up survey was conducted from April 2016 to December 2016 and was completed by 2286 participants (86.46% follow-up rate). Cox regression was used to evaluate the association of each component and the number of combinations of MS components on the development of CVD.

**Results:**

A total of 278 CVD patients were enrolled from rural residents of Xinjiang. The average age of the MS and non-MS groups was 46.33 and 38.71 years, respectively. Independent associations with CVD were found for elevated blood pressure (BP) (adjusted hazard ratio (HR) [aHR] = 1.50,95%confidence interval [CI]: 1.08–2.08), elevated waist circumference (WC) (aHR = 1.60, 95%CI: 1.19–2.15), and elevated triglycerides (TG) (aHR = 1.44, 95%CI: 1.04–2.01). Participants with one to 5 MS components had an increased HR for developing CVD, from 1.82to 8.59 (P for trend < 0.001), compared with those with no MS components. The risk of developing CVD increased when TG and WC coexisted (aHR = 2.16, 95%CI: 1.54–3.04)), when TG and BP coexisted ((aHR = 1.92, 95%CI: 1.32–2.79), and when WC and BP coexisted (aHR = 1.93, 95%CI: 1.33–2.82)). However, no significant interactions were found between BP, WC, and TG.

**Conclusions:**

Elevations of BP, WC, and TG were independent risk factors for CVD in Kazakhs. Control of these factors is important to prevent CVD in this population.

## Background

Cardiovascular disease (CVD) has become the leading cause of death in China [1]. Although medical treatments are available for CVD, they are not a lasting solution and the long-term harmful impact of the disease on patient health is challenging to treat. Prevention of CVD is thus an urgent public health issue. However, the underlying cause of CVD is unknown, posing a danger for individuals at high risk of developing the disease.

Metabolic syndrome (MS) is a highly prevalent constellation of vascular risk factors that include elevated blood pressure (BP), elevated blood glucose, obesity, and dyslipidemia [2]. A meta-analysis [3] that included 87 different studies found that MS was associated with an increased risk of CVD (relative risk: 2.35; 95% confidence interval [CI]: 2.02–2.73). Several studies have demonstrated the association of MS with CVD among Asian populations in Hong Kong [4], Japan [5], mainland China [6], and Taiwan [7]. However, the differential clustering of MS components and their association with CVD in a Kazakh population in Xinjiang province of China is not clearly understood. Whether this population is most at risk for CVD development in the presence of MS is unclear. Accordingly, there is a need for studies on risk of CVD in the Kazakh population to identify effective methods of disease prevention for these individuals.

Xinjiang, a province in northwestern China, is a multiethnic settlement that includes nomadic Kazakh individuals. Owing to their special ethnicity, living environment, and genetic characteristics, there is a growing double epidemic of dyslipidemia and obesity in Kazakhs [8–10]. To our knowledge, there have been no studies investigating the association of MS with the risks of CVD in the Kazakh population due to the limited public health care resources and poor transportation in the rural regions of Xinjiang. Therefore, the objective of this study was to examine the association of MS and its components with CVD, to further prevent and control the CVD in Kazakhs.

## Methods

### Study population

**Fig. 1:**
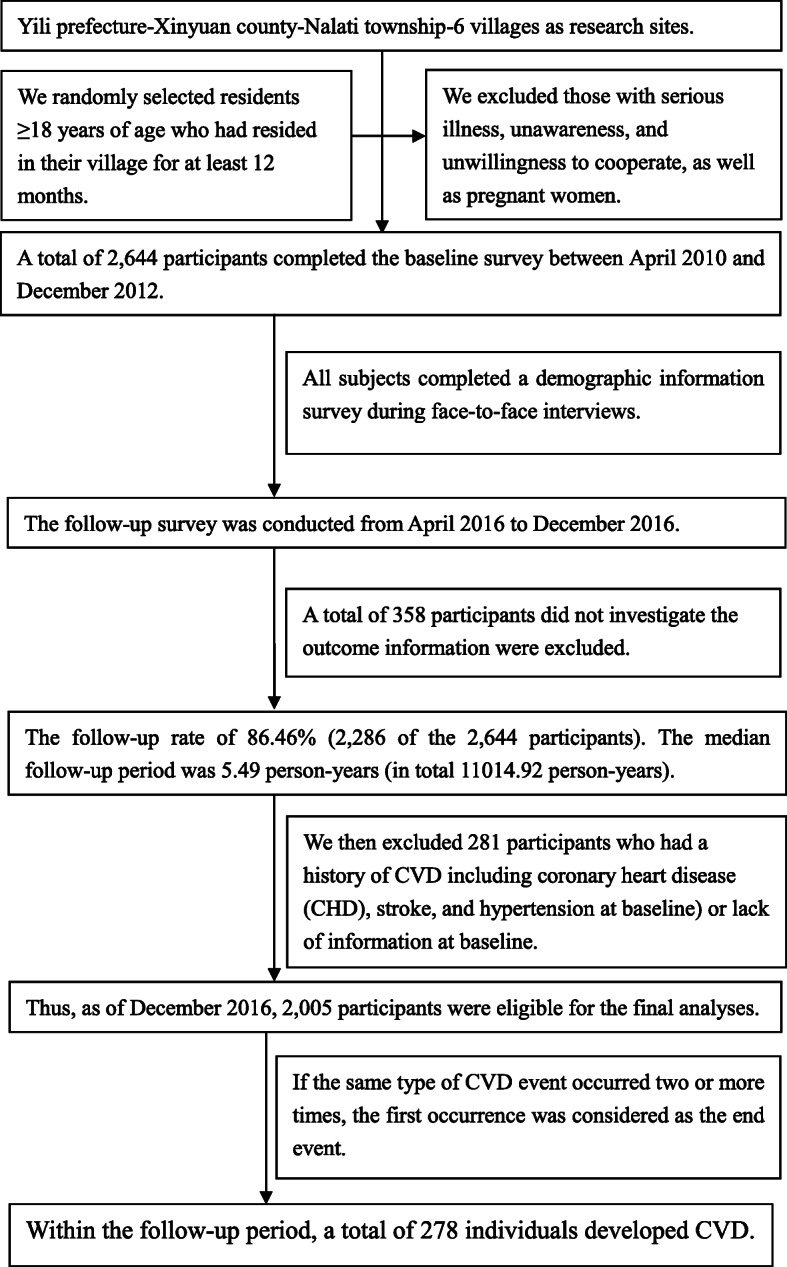
Flowchart of the study

A four-stage cluster random sampling was used to obtain a representative sample. In the first stage, according to the geographical distribution of the minority populations in Xinjiang, we selected Yili as the representative prefecture. Yili is approximately 4407 km (2739 miles) from Beijing. Approximately 98% of the population belong to the Kazakh minority. In the second stage, we randomly selected Xinyuan County from the Yili Prefecture. In the third stage, we randomly selected sex villages from Xinyuan County. In the final stage, we randomly selected residents ≥18 years of age who had resided in their village for at least 12 months. We excluded those with serious illness, unawareness, and unwillingness to cooperate, as well as pregnant women. A total of 2644 participants completed the baseline survey between April 2010 and December 2012. To improve the rate of follow-up, subjects were interviewed in-person and in household surveys. The follow-up survey was conducted from April 2016 to December 2016. The follow-up rate of 86.46% (2286 of the 2644 participants). The median follow-up period was 5.49 person-years (in total 11,014.92 person-years). We then excluded 281 participants who had a history of CVD including coronary heart disease (CHD), stroke, and hypertension at baseline. Thus, as of December 2016, 2005 participants were eligible for the final analyses. Within the follow-up period, a total of 278 individuals developed (Fig. 1). The person-years were calculated as the sum of the individual follow-up times until the occurrence of a CVD incident or the end of 2016.

### Epidemiological survey and biochemical analyses

All subjects completed a demographic information survey during face-to-face interviews. Detailed information about diet, drinking, details of existing disease, and family history of disease was collected by trained investigators. Trained staff measured weight, height, waist circumference (WC), and BP according to standard methods [11]. Blood samples were drawn from the cubital vein into tubes containing heparin sodium in the morning after an 8-h fast. Blood samples were cryopreserved before being transported. Fasting plasma glucose (FPG), triglyceride (TG), and high density lipoprotein cholesterol (HDL-C) levels were measured using a biochemical auto-analyzer (Olympus AU 2700; Olympus Diagnostics, Hamburg, Germany) in the clinical laboratory of the First Affiliated Hospital of Shihezi University School of Medicine. All described methods were performed according to the approved guidelines and regulations. All participants signed informed consent forms before joining the study. This study was approved by the Institutional Ethics Review Board (IERB) of the First Affiliated Hospital of Shihezi University School of Medicine (IERB No.:SHZ2010LL01).

### Diagnostic criteria for CVD

Participants who had first-ever occurrence of stroke (ischemic or hemorrhagic), CHD, or hypertension during the follow-up period were diagnosed as having CVD. Subjects with CHD were hospitalized during the follow-up period if coronary intervention (cardiac catheterization or coronary bypass surgery) was performed, if there was angina (or nitroglycerin was initiated after cohort study), if myocardial infarction occurred, and because of congestive heart failure. Data regarding CVD events were obtained from the hospital medical records and questionnaire responses. If the same type of event occurred two or more times, the first occurrence was considered as the end event. CVD were determined based on self-reported questionnaire responses, medical insurance records, and local hospital discharge records from 2010 to 2016. Patients with self-reported manifestations of CVD findings required a certificate of diagnosis from a medical institution in their township during the investigation.

### Definition of MS

According to the third report describing treatment of adult Americans as part of a modified United States National Cholesterol Education Program (2005 NCEP-ATP III) [2].MS is defined when three or more factors are met. These factors include: (1) central obesity: WC ≥90 cm for men and ≥ 80 cm for women; (2) elevated TG level ≥ 150 mg/dL (1.70 mmol/L); (3) reduced HDL-C < 40 mg/dL(1.03 mmol/L) for men and < 50 mg/dL(1.30 mmol/L) for women; (4) elevated BP ≥130/85 mmHg, or have received the appropriate treatment for hypertension or had been previously diagnosed with hypertension; and (5) elevated FPG ≥100 mg/dL(5.6 mmol/L) or had received appropriate treatment for diabets or had been previously diagnosed with type 2 diabetes.

### Confounding factors

Traditional risk factors used in the data analysis included age, sex, drinking (drinker/non-drinker), and family history of hypertension, diabetes, and CVD.

### Statistical analyses

The database construction and some statistical analyses have been previously described [12]. Cox proportional hazards regression model was used to evaluate the association of each component and the number of combinations of MS components on the development of CVD. Next, we evaluated the interactions between TG and WC, TG and BP, WC and BP. The grouping method and additive interactions have been previously described [12]. Multiplicative interactions among BP, WC, and TG were evaluated by incorporation of the dummy variable into a Cox regression model. All statistical analyses were performed using SPSS version 17.0 for Windows (SPSS Inc., Chicago, IL,USA). All statistical tests were two-sided, and *P* <  0.05 was considered statistically significant.

## Results

### Baseline characteristics of subjects

Table 1summarizes the baseline characteristics. In this study, 2286 people completed the follow-up and the median follow-up period was 5.49 person-years (in total 11,014.92 person-years). During the follow-up,278 who had their first CVD event were identified. The average age of the MS and non-MS groups was 46.33 and 38.71 years, respectively. There were 330 females and 198males in the MS group, and847 females and 630males in the non-MS group. The prevalence of MS was 23.10% (528/2286). Those with MS were significantly older and had higher WC, systolic BP, diastolic BP, TG, and FPG than those without MS. Prevalence of smoking and drinking was significantly higher in the MS group than in the non-MS group (Table 1).

**Table 1:** Baseline general characteristics of subjects

Risk factor	MS	Non-MS	*P*
Sex, female / male	330 / 198	847 / 630	0.039
Age, years	46.33 ± 12.04	38.71 ± 11.83	< 0.001
WC, cm	91.24 ± 10.67	79.40 ± 9.36	< 0.001
SBP, mmHg	135.74 ± 18.78	122.97 ± 17.95	< 0.001
DBP, mmHg	87.42 ± 12.91	78.71 ± 11.94	< 0.001
HDL-C, mmol / L	1.21 ± 0.38	1.41 ± 0.54	< 0.001
TG, mmol / L	1.79 ± 1.56	1.06 ± 0.96	< 0.001
FPG, mmol / L	5.75 ± 1.66	5.01 ± 0.93	< 0.001
Smoking rate, *n* (%)	195 (36.93)	434 (29.38)	0.001
Drinking rate, *n* (%)	80 (15.15)	147 (9.95)	0.001
Family history of hypertension, *n* (%)	206 (39.02)	501 (33.92)	0.035
Family history of diabetes, n (%)	9 (1.70)	9 (0.61)	0.022
Family history of CVD, *n* (%)	56 (10.61)	113 (7.65)	0.036
Incidence of CVD, *n* (%)	114 (21.59)	164 (11.10)	< 0.001

*WC* waist circumference, *SBP* systolic blood pressure, *DBP* diastolic blood pressure, *HDL-C* high-density lipoprotein cholesterol, *TG* triglyceride, *FPG* fasting plasma glucose, *MS* metabolic syndrome, *CVD* cardiovascular diseases

### Adjusted hazard ratio (aHR) for CVD and MS with its components in cox proportion hazard regression model

As was expected, MS and the individual components of MS were significantly associated with the risk of developing CVD, independent of age, sex, drinking, and family history of hypertension, diabetes, and CVD. After adjusting for four other MS components and the traditional risk factors above, the elevated BP (aHR 1.50; 95% CI: 1.08–2.08), elevated WC (aHR 1.60; 95% CI: 1.19–2.15) and elevated TG (aHR 1.44; 95% CI: 1.04–2.01) were still independently associated with CVD (Table 2).

**Table 2:** Adjusted HR for CVD and MS with its components in Cox proportion hazard regression model

Risk factor	Population exposure ratio, (%)	Incidence of CVD, (%)	HR (95%CI)^a^	aHR (95%CI)^b^	aHR (95%CI)^c^
MS	26.33	21.59	2.22 (1.75–2.83)	1.46 (1.14–1.87)	
WC	43.99	20.07	2.28 (1.78–2.91)	1.59 (1.23–2.05)	1.60 (1.19–2.15)
BP	45.89	19.57	2.07 (1.61–2.64)	1.49 (1.11–2.00)	1.50 (1.08–2.08)
TG	19.75	18.69	1.78 (1.36–2.32)	1.55 (1.19–2.03)	1.44 (1.04–2.01)
FPG	28.08	16.70	1.33 (1.04–1.70)	1.07 (0.83–1.38)	1.14 (0.86–1.53)
HDL-C	40.00	15.09	1.48 (1.17–1.88)	1.36 (1.07–1.74)	1.34 (0.99–1.82)

*HR* hazard ratio, ^a^ univariate analysis; ^b^ adjusted for age, sex, drinking, and family history of hypertension, diabetes, and *CVD* ^c^ adjusted for four other MS components and the age, sex, drinking, and family history of hypertension, diabetes, and CVD.

### aHR of number of MS components associated with CVD

In contrast with the participants with no MS components, the incidence of CVD was increased with the increase in the number of abnormal components of MS, ranging from 5.56 to 37.50%.Participants with one to five MS components had an increased HR for developing CVD, from 1.82 (95%CI: 1.07–3.11) to 8.59 (95%CI:4.43–16.67) (trend *P* < 0.001), compared to those with no MS components. This trend persisted even after adjusting for age, sex, drinking, and family history of hypertension, diabetes, and CVD (Table 3).

**Table 3:** Adjusted HR of number of MS components associated with CVD

	CVD (n)	Incidence of CVD, (%)	HR (95%CI)	aHR** (95%CI)
0 component	17	5.56	1.00 (reference)	1.00 (reference)
1 component*	62	10.25	1.82 (1.07–3.11)	1.49 (0.87–2.55)
2 components*	85	15.02	2.86 (1.70–4.82)	1.76 (1.03–2.99)
3 components*	60	17.96	3.58 (2.09–6.13)	1.89 (1.08–3.29)
4 components*	36	24.66	5.68 (3.19–10.12)	2.56 (1.41–4.65)
5 components*	18	37.50	8.59 (4.43–16.67)	4.25 (2.16–8.39)
P for trend			< 0.001	< 0.001

*Compared with those with 0 components of MS; HR, univariate analysis; **adjusted for age, sex, drinking, and family history of hypertension, diabetes, and CVD; Abbreviations are defined in Tables 1 and 2

### Analysis of interactions between BP, WC and TG associated with CVD

We evaluated the effect of the interaction between WC and TG on CVD development. These two separate factors, arranged into four different subgroups according to their individual absence or presence, were analyzed in pairs by a Cox regression model. Relative to the subgroup without TG and without WC, the HRs associated with CVD were 1.45 (95% CI: 1.08–1.94), 1.27 (95% CI: 0.74–2.21), and 2.16 (95% CI: 1.54–3.04) for the subgroups without TG and with WC, with TG and without WC, and with TG and WC, respectively (Table 4). Moreover, with regard to the indexes of additive interaction, the RERI value was 0.53 (95%CI, − 0.40-1.46), AP was 0.25 (95%CI, − 0.15-0.65) and SI was 1.91 (95%CI, 0.47–7.70), which indicated no additive interaction between the two risk factors. Interaction analysis between TG or WC and BP on CVD development revealed no additive interactions (Table 5).

**Table 4:** Multiplicativeinteractions analysis among BP, WC, and TG associated with CVD

Factor1	Factor2	CVD (*n*)	Incidence of CVD, (%)	aHR (95%CI)
WC	TG			
+	+	59	23.79	2.16 (1.54–3.04)
+	–	118	18.61	1.45 (1.08–1.94)
–	+	15	10.14	1.27 (0.74–2.21)
–	–	86	8.82	1.00 (reference)
BP	TG			
+	+	52	25.00	1.92 (1.32–2.79)
+	–	128	17.98	1.22 (0.90–1.66)
–	+	22	11.70	1.49 (0.93–2.40)
–	–	76	8.47	1.00 (reference)
BP	WC			
+	+	122	24.90	1.93 (1.33–2.82)
+	–	55	14.03	1.40 (0.93–2.11)
–	+	58	13.49	1.86 (1.24–2.79)
–	–	43	6.20	1.00 (reference)

*aHR* adjusted for age, sex, drinking, family history of hypertension, diabetes, and CVD; Abbreviations are defined in Tables 1 and 2.

**Table 5:** Additive interactions among BP, WC, and TG

	Relative excess risk		Attributable proportion		Synergy index	
	Point estimation	95%CI	Point estimation	95%CI	Point estimation	95%CI
WC and TG	0.53	(−0.40–1.46)	0.25	(−0.15–0.65)	1.91	(0.47–7.70)
BP and TG	0.31	(−0.66–1.27)	0.15	(−0.30–0.60)	1.42	(0.43–4.70)
BP and WC	0.03	(−0.89–0.94)	0.01	(−0.38–0.40)	1.02	(0.51–2.03)

Abbreviations are defined in Tables 1 and 2.

## Discussion

Numerous studies have suggested that MS is a cluster of CVD risk factors [13–16]. A meta-analysis [3] that included 87 different studies found that MS was associated with an increased risk of CVD (relative risk: 2.35; 95% CI: 2.02–2.73). The predictive value of MS and its contribution to CVD should be ascertained in region-specific populations [17], considering that its effects have been studied in select populations [18]. This study involved Kazakhs. The dietary of this ethnic group is similar, and there is not much variation. They eat pasta and drink milk tea every day. And they don’t intermarry with other ethnic groups, so their eating habits remain unchanged. Their special ethnicity, living environment, and genetic characteristics contribute to the higher prevalence of MS and related diseases in Kazakhs than in other ethnic groups [8–10]. However, there are scant longitudinal data regarding the predictive value of MS in the Kazakh population. In this study, MS was associated with CVD in Kazakh subjects. The increased risks of CVD remained significant after adjusting for general risk factors. These findings were also reported in some earlier studies [19–22]. These results imply a significant role of MS in the development of CVD in this population. However, another study did not find a no significant correlation between MS and CVD in a biracial cohort of Whites and Blacks, even after adjusting for risks associated with MS components [23]. This finding was also highlighted in the West of Scotland Coronary Prevention Study [24]. These discrepancies may be explained in part by the different MS definitions used and the prevalence of individual components of MS in the studied populations.

Presently, each component of MS appeared to be was associated with an increased prospective risk of CVD. Furthermore, as the number of MS components increased, the risk of CVD also increased, with a significant and cumulative-component response trend. These findings suggest the presence of a cumulative effect of MS components in the elevated risk of CVD. These are important findings because the relationships between clustering patterns of MS and CVD risk have not been thoroughly characterized in Kazakh populations. This synergistic association is also noteworthy as it provides valuable information for the establishment of appropriate policies in preventive CVD for the inhabitants of Xinjiang. This linear synergistic correlation has been previously reported in other ethnic groups [21, 22].

The independent association of MS and its components in predicting CVD has become a subject of recent research interest. However, which MS component is more strongly associated with CVD remains unclear. In the Asia Pacific region, up to 66% of some subtypes of CVD can be attributed to hypertension [25]. A more important role for BP than other components in determining cardiovascular events has been indicated in several studies [26, 27]. Likewise, after adjusting for four other MS components as well as general risk factors, BP remained independently associated with increased risks of CVD in the Kazakh population. The collective data indicate that elevated BP is an independent risk factor for the development of CVD.

Current research found the elevations of BP, WC, and TG were independent risk factors for CVD in the Kazakh population. Suh et al. [16] found that BP and abdominal obesity were key predictors of CVD in Koreans when adjusting for general risk factors and MS components. For subjects in the National Health and Nutrition Examination Survey III, BP and HDL-C were associated with CHD when adjusted for general risk factors and MS components [28]. Hadaegh et al. [29] studied Middle-East Caucasian residents in Tehrani and highlighted that the FPG level in women and WC in men were independently associated with CVD. These reports are inconsistent in terms of the MS components that predict CVD. These discrepancies may be explained in part by the different study populations, follow-up periods, MS definition used, and prevalence of individual components of MS in different populations.

Hypertension is a risk factor for the development of atherosclerosis. Both clinical and experimental data show that elevated BP enhances the development of atherosclerosis. In fact, atherosclerosis tends to occur only in those parts of the vascular system subjected to high pressure [30]. The elevated TG levels are independently associated with an increased risk of CVD [31]. Elevated blood levels of triglyceride remnants have been linked to the progression of CVD by directly contributing to atherosclerotic plaque formation and progression [32, 33]. Both BMI and WC are strongly correlated with total body adipose tissue mass with a correlation coefficient (r) > 0.80 [34]. The WC is a better correlate of intra-abdominal adipose tissue (r = 0.77–0.79) than BMI (r = 0.59–0.69) [34]. Adipose tissue functions as an endocrine organ by secreting multiple immune-modulatory proteins known as adipokines. Obesity leads to increased expression of pro-inflammatory adipokines and diminished expression of anti-inflammatory adipokines, resulting in the development of a chronic low- grade inflammatory state. This adipokine imbalance is thought to be a key event in promoting both systemic metabolic dysfunction and cardiovascular disease [35].

We analyzed further the interaction between TG and WC or between WC / TG and BP and explored whether their coexistence was an additional risk factor for CVD. When TG and WC coexisted, the aHR was 2.16, which indicated that the accumulation of TG and WC strengthens the CVD risk. Therefore, it is plausible that the coexistence of TG and WC constitutes the highest CVD risk. If so, the growing double epidemic of obesity and dyslipidemia among Kazakh people may greatly and rapidly increase their burden of developing CVD. Therefore, the dietary intervention play an important role in decreasing the incidence of CVD and improving the quality of life in the Kazakh population.

### Study limitations

This study is a large-scale prospective cohort study of a Kazakh population with a long follow-up period that reports the association of MS with CVD incidence. Our study had some limitations. First, the diagnosis of MS was based on a single measurement of its components at baseline, as was the case in other epidemiological studies [36]. Second, because of site-specific limitations, the CVD outcomes analyzed here failed to include hospitalization or outpatient visits due to a transient myocardial ischemia that prevented symptom remission, which may have led to the underestimation of CVD incidence. Third, Kazakh is a large nomadic nation in Xinjiang and the fluidity is relatively large, physical activity was not assessed and therefore not accounted for in the multivariate model. Despite the limitations, our findings may be generalized to populations residing in low-income rural areas of Xinjiang. In addition, our findings may provide some important insights regarding issues related to the relationship between MS and CVD in rural Kazakh populations living in other countries, such as Kazakhstan and Uzbekistan owing to similarities in religion, culture, lifestyle, diet, and genetic background in these ethnic groups.

## Conclusions

Elevated BP, elevated WC, and elevated TG are independent risk factors for CVD in Kazakhs. Therefore, it is important to control BP, WC, and TG to prevention CVD in this population.

## Data Availability

The datasets used and analyzed during the current study are available from the corresponding author on reasonable request.

## Abbreviations

CVD: Cardiovascular disease
MS: Metabolic syndrome
CHD: Coronary heart disease
WC: Waist circumference
BP: Blood pressure
SBP: Systolic blood pressure
DBP: Diastolic blood pressure
FPG: Fasting plasma glucose
TG: Triglyceride
HDL-C: High density lipoprotein cholesterol
IERB: Institutional Ethics Review Board
WC (−): Without WC
BP(−): Without BP
TG(−): Without TG
WC (+): With WC
BP (+): With BP
TG (+): With TG
RERI: Relative excess risk due to interaction
AP: Attributable proportion due to interaction
SI: Synergy index
CI: Confidence interval
HR: Hazard ratio
